# The Dominant Eye: Dominant for Parvo- But Not for Magno-Biased Stimuli?

**DOI:** 10.3390/vision4010019

**Published:** 2020-03-12

**Authors:** Brian K. Foutch, Carl J. Bassi

**Affiliations:** 1 Rosenberg School of Optometry, University of the Incarnate Word, San Antonio, TX 78209, USA; 2 College of Optometry, University of Missouri-St Louis, St. Louis, MO 63121, USA; bassi@umsl.edu

**Keywords:** dominant eye, contrast, parvocellular, magnocellular

## Abstract

Eye dominance is often defined as a preference for the visual input of one eye to the other. Implicit in this definition is the dominant eye has better visual function. Several studies have investigated the effect of visual direction or defocus on ocular dominance, but there is less evidence connecting ocular dominance and monocular visual thresholds. We used the classic “hole in card” method to determine the dominant eye for 28 adult observers (11 males and 17 females). We then compared contrast thresholds between the dominant and non-dominant eyes using grating stimuli biased to be processed more strongly either by the magnocellular (MC) or parvocellular (PC) pathway. Using non-parametric mean rank tests, the dominant eye was more sensitive overall than the non-dominant eye to both stimuli (z = −2.54, *p* = 0.01). The dominant eye was also more sensitive to the PC-biased stimulus (z = −2.22, *p* = 0.03) but not the MC-biased stimulus (z = −1.16, *p* = 0.25). We discuss the clinical relevance of these results as well as the implications for parallel visual pathways.

## 1. Introduction

Many eye care professionals still assume pure dominance laterality in their patients; that is, in lieu of some test for eye-dominance, clinicians often simply assign ocular mastery to the right eye of right-handed patients and to the left eye for left-handed patients. This habit exists despite almost a century of evidence that eye dominance is unrelated to cerebral laterality or hand dominance [1,2,3,4,5,6]. This debate has been extended to the overall relevance of the dominant eye. Sheard demonstrated nearly a century ago that the dominant eye has less tolerance for blur than the non-dominant eye [7]. However, the leading opinions were that ocular dominance only existed in cases of asymmetrical visual acuity resulting from ocular pathology or anisometropia, and the entire “theory of ocular dominance had no real significance beyond determining the sighting eye for sport” [8,9].

The clinical relevance of the dominant eye is now well-accepted, as several studies have since verified the existence of physiological, sensory and motor bases for ocular dominance in patients with normal binocularity and equal refractive errors [10,11,12,13,14,15,16,17,18]. For example, Hofeldt concluded that placing a moderate neutral density filter over the dominant eye diminished motion task performance more than placing the filter over the non-dominant or both eyes [12]. These studies suggest that one eye at least guides visual direction, and it is safe to presume that eye should be assigned as the better seeing eye when balancing refractions or assigning distance and near eyes in monovision contact lens prescriptions.

Although some of these studies compared monocular visual thresholds between the dominant and non-dominant eyes, only Kommerrell et al. constrained any of their analysis to a motor—or sighting—definition of the dominant eye [15].

Several other sensory tests, including letter-strength (non-overlapping and overlapping) [17], letter-polarity [18], phase combination or phase suppression [19], motion strength [20] and motion rivalry [21], have been used to measure eye dominance. Bossi et al. [22] compared these measures and concluded that letter-polarity had the most intra-observer consistency and inter-observer discriminatory power when comparing these estimates to stereoacuity [23].

There are numerous processes involved in transforming visual sensations into the perception of brightness, color and forms. In humans and other primates, physiological and behavioral evidence indicate two anatomically and functionally distinct pathways originating in the magnocellular (MC) and parvocellular (PC) retinal ganglion cells [24,25]. Object location, movement, low contrast sensitivity and global analysis of visual scenes are processed more efficiently through the MC pathway, whereas object and pattern recognition as well as color (in particular, red–green opponency) are processed more efficiently through the PC pathway [26,27,28,29]. The MC and PC retinal ganglion cells project to the dorsal portion of the lateral geniculate nucleus (dLGN) of the thalamus, and the dLGN projects to cortical areas involved in visual processing, chiefly to the primary visual cortex (V1). These MC and PC pathways can be followed deep into the visual processing areas of the posterior parietal (PP) and inferior temporal (IT) cortex and are thought by some to form anatomically distinct dorsal (MC) and ventral (PC) processing streams [30].

There is general agreement concerning the existence and clinical relevance of these parallel pathways [31]. The persistent debate is whether individual differences in parallel pathways lead to measurable differences in visual processing. What if those differences are intra-observer, comparing function between the dominant and non-dominant eyes? A paradigm used by Zhou et al. [32] to induce temporary eye dominance by depriving one eye of visual input found that eyes could have a different dominance for chromatic vs. achromatic, depending on the exact stimulus used. However, to our knowledge, the current study is the first attempt to compare responses of the dominant and non-dominant eyes to stimuli biased specifically toward parvocellular (PC) or magnocellular (MC) processing. While their findings predate seminal investigations into sustained (i.e., PC) and transient (i.e., MC) visual detectors [33], classic clinical investigations revealed that the dominant eye determines visual direction [34] or is more sensitive to lens-induced blur when viewing binocularly [7,35]. Based on these findings, we expected responses of the dominant eye to be more sensitive to the foveal, stationary (PC-biased) target. Our inclusion of a transient, low-contrast (MC-biased) stimulus was based on electrodiagnostic [36] and visual field [37] studies that revealed interactions between MC and PC contributions to visual functioning. However, at least one study has shown that reducing contrast with a neutral density filter placed over the dominant eye diminished binocular motion task performance more than when placed over the non-dominant eye [12]. Thus, we expected that the dominant eye would also be more sensitive to the transient, low-contrast (MC-biased) target.

## 2. Materials and Methods

### 2.1. Subjects

Overall, 28 subjects (17 females and 11 males) participated, and their ages ranged from 21 to 38 years. Inclusion criteria included visual acuity corrected to at least 20/20 in each eye, near stereoacuity of at least 1 min of arc, no color vision defects as measured by Ishihara pseudo-isochromatic plates and no history of ocular disease or surgeries that may affect contrast sensitivity thresholds. The protocol was approved by the Institutional Review Board at the University of Missouri—St. Louis, and informed consent was obtained from each subject.

### 2.2. Determining the Dominant Eye

Walls discussed several methods to determine the motor dominant (sighting) eye and concluded that Bryngelson’s modification of the Dolman method was the most satisfactory [38]. This technique is often referred to as the “hole in the card” method and is described as follows: A 30 cm square rigid board with a 3 cm hole in the geometric center was held in both hands and placed in the lap. Each subject was asked to raise the board with arms fully extended and fixate a small (2 cm) circular target on a computer screen placed 2.5 m away. By alternate occlusion, the experimenter determined which eye was fixating the target. This was repeated for a total of three trials each with arms fully extended, with arms partially flexed (as one would read a book) and with the arms fully flexed with the board only a few centimeters from the nose. This entire procedure was repeated with the subject being asked to lower the board from above the target. The eye used to fixate the majority of all the 18 trials was determined to be the dominant eye.

All subjects fixated at least 17 of the 18 times with the same eye, except for one female subject who used the right eye in 10 of the 18 trials and the left eye in 8 of the 18 trials. Since the dominant eye used in this study was defined as the eye that guided visual direction, this subject’s results were excluded from the analysis.

### 2.3. Stimuli

**Figure 1:**
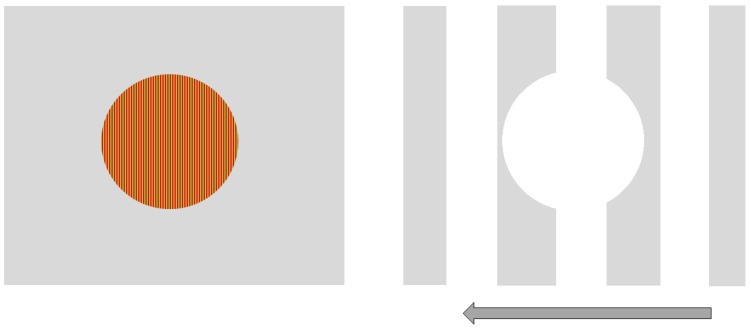
Parvocellular (PC)-biased (**left**) and magnocellular (MC)-biased (**right**) stimuli used in the study.

We used Vision Works 4.0 Contrast Sensitivity software (Vision Research Graphics, Inc.; Durham, NC) to generate and display sinusoidal gratings on a 21” monitor located 250 cm from the observer. Two stimuli were designed to be processed more strongly either by the magnocellular (MC) or parvocellular (PC) pathway. Our goal for the MC-biased target was for it be larger, have a low spatial frequency, non-foveal, achromatic and involve flicker or movement. Therefore, the MC-biased target was rectangular and subtended a visual angle of 5 degrees wide by 3 degrees tall at a 250 cm viewing distance. The stimulus remained stationary, but a black and white grating pattern of 1 cycle/degree (cpd) drifted quickly to the left at 35 cycles/s. In addition, there was a circular portion removed from the center of the grating. This center-surround configuration was designed to further bias (non-foveal) stimulation of the MC visual pathway. Conversely, the PC-biased stimulus was a small, central, stationary red on green circular target subtending 1.25 degrees of the visual angle and contained a higher spatial frequency (20 cycle/degree) grating. Both stimuli were presented against an achromatic background that varied as the average luminance of the presented stimulus (see Figure 1).

### 2.4. Measuring and Analyzing Contrast Thresholds

Each eye was tested, and contrast thresholds were obtained for each stimulus using a QUEST modified staircase procedure [39]. The order of testing was randomized for which eye and stimulus was tested first. Subjects were also instructed that their reaction times would be analyzed, but the primary experimental task was whether they could resolve the grating stimulus against the background. Consequently, reaction times widely varied and were of little use in the analysis. Contrast thresholds were not distributed normally. However, as the contrast threshold comparisons between the eyes and between stimuli were intra-observer and cannot be assumed to be independent, we analyzed them by Wilcoxon signed-rank mean tests. Stimulus type (MC- vs. PC-biased), eye (dominant vs. non-dominant) and gender were all considered in the analysis. All statistical analyses were performed using SPSS (IBM, Chicago, IL, USA).

## 3. Results

**Figure 2:**
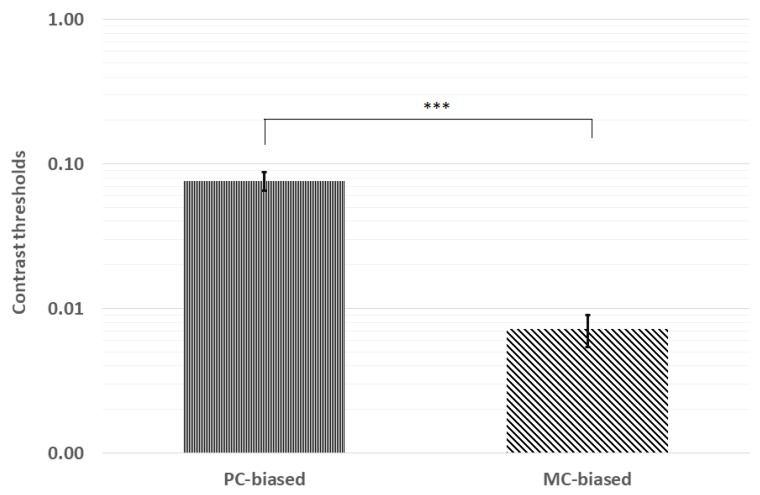
Contrast thresholds were significantly lower for the MC-biased stimulus than the PC-biased stimulus. *** *p* < 0.001; error bars represent ±1 standard deviation.

Contrast thresholds were first analyzed for normality via Kolmogorov–Smirnov tests. Pooled contrast thresholds were not distributed normally (*p* < 0.001), and neither were thresholds for PC (*p* = 0.046) nor MC (*p* < 0.001) stimuli. Therefore, we analyzed mean contrast thresholds using the Wilcoxon signed-rank test. The mean ranks indicated significantly higher thresholds for the PC-biased (28.8) stimulus than for the MC-biased (10.5) stimulus (z = −6.42, *p* < 0.001; see Figure 2).

**Figure 3:**
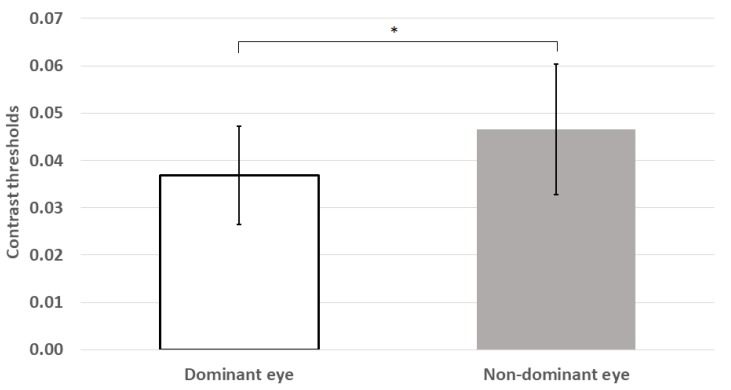
Mean contrast thresholds were significantly lower for the dominant eye than those for the non-dominant eye. * *p* < 0.05; error bars represent ±1 standard deviation.

Mean thresholds were also significantly higher for the non-dominant eye than the dominant eye (z = −2.54, *p* = 0.011; see Figure 3).

There was also a trend toward lower thresholds in female subjects, but these differences have been explored more fully and reported elsewhere [40].

**Figure 4:**
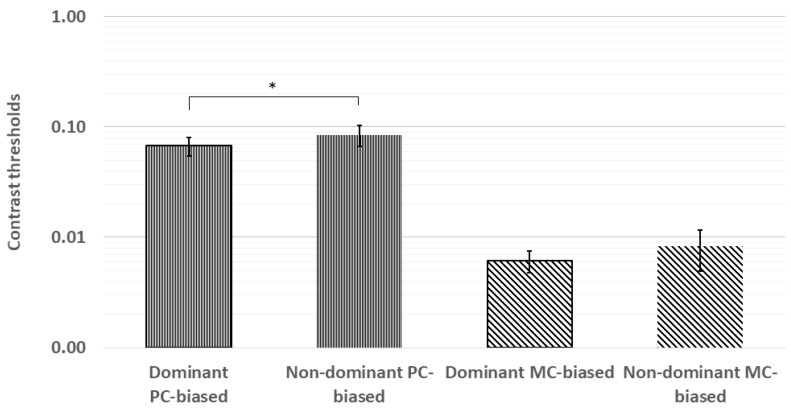
The planned paired comparisons of contrast thresholds revealed significantly lower thresholds in the dominant eye for the PC-biased stimulus but not for the MC-biased stimulus. * *p* < 0.05; error bars represent ±1 standard deviation.

The planned paired comparisons of contrast thresholds revealed significantly lower thresholds in the dominant eye for the PC-biased stimulus (z = −2.22, *p* = 0.03) but not for the MC-biased stimulus (z = −1.16, *p* = 0.25). These results are shown in Figure 4.

## 4. Discussion

There is a wealth of evidence supporting the existence of two distinct parallel visual pathways—magnocellular and parvocellular—tuned separately for different types of visual inputs [27,31,41,42,43]. The magnocellular pathway is tuned to detect “where” things are and is more sensitive to transient, low contrast, low spatial frequency, achromatic targets. The parvocellular system is tuned to detect “what” things are and is therefore more sensitive to stationary, high spatial frequency, chromatic targets. As the reference point for visual direction is the fovea (see [44] for a sample reference), it is then not surprising that monocular performance for the small, red-green stimulus was better using the dominant or sighting eye. This result is consistent with previous findings revealing superior functioning in the dominant eye under binocular viewing conditions [11,12,16,45] and may further suggest that PC-biased objects seen through the dominant eye are in some way more discernible than those seen by the non-dominant eye. It has also been suggested that higher discernibility leads to faster processing of the visual information arriving from the dominant eye [45]. However, faster processing should result in better magnocellular processing in the dominant eye, which was not found in the current study.

We need to acknowledge two factors that limit inferences from our results, particularly for the non-significant findings for the MC-biased stimulus. First, a sample size of 28 subjects is very small. Secondly, we experienced a floor effect for the MC-biased stimulus. These concerns will be addressed together. To approximate experiment time and determine initial contrast values for the QUEST algorithm, pilot data were collected from eight subjects. We determined an effect size of d = 0.45 for the PC-biased stimulus but only d = 0.15 for the MC-biased stimulus. Working on the prediction that contrast thresholds would be lower for the dominant eye for both stimuli, a one-tailed power analysis required us to recruit over 100 subjects to detect an advantage in the dominant eye for the MC-biased stimulus. Given a fixed timeframe for data collection and lack of direct compensation for the subjects, this was not practical. At that time, the mean contrast threshold for the MC-biased stimulus was approximately 0.01. In the hopes of increasing the power of the comparison, we further optimized the stimulus to reduce contrast thresholds to approximately 0.005. In doing so, we unknowingly experienced a floor effect for the MC-biased stimulus, as the contrast limit of the system was 0.004. This floor effect has limited our inferences concerning the MC-biased stimulus.

We also limited our subject pool to those with complete right (R) or left (L) dominance. If we had not done so, we could have regressed a continuous dominance level on contrast thresholds. While restricting our definition to dichotomous (R v. L) dominance somewhat limited our analysis, a natural follow-up could assess the impact of temporarily depriving one eye (as in [32]) with similarly MC- and PC-biased stimuli to further delineate the separate effects on each pathway. In effect, could one train the non-dominant eye—via a temporary form or red/green chromatic deprivation of the dominant eye—to obtain lower thresholds to PC-biased stimuli? Likewise, could we train better low contrast thresholds to flickering stimuli in the non-dominant eye via complete occlusion of the dominant eye? In doing so (i.e., balancing dominance between the two eyes), it would be theoretically possible to increase activity in binocular cortical areas (i.e., V1 and neighboring areas) that are limited by ocular dominance. Quantifying these effects would be challenging and expensive, requiring fMRI during binocular viewing. However, this is an interesting area for a follow-up study.

Additional areas for future study could include more direct investigations into moving (not simply drifting) gratings, which would also tap more directly into the “where” aspects of MC processing. One could also use flicker rate thresholds as surrogates for MC functioning. In the current investigation, we determined contrast thresholds using a suprathreshold drifting speed (35 Hz). One could easily flip that paradigm and determine critical flicker rates at fixed suprathreshold contrast levels. For the PC-biased stimulus, one could also fix contrast and determine threshold spatial frequency thresholds. These adjustments would further delineate the aspects of MC and PC pathways. Lastly, as the higher order aspects of ventral (or PC) streams have been implicated in increased brightness to luminance (B/L) ratios for certain color stimuli [46], it would be interesting to determine if the B/L ratios are different between dominant and non-dominant eyes.

The current results simply suggest that the eye that is used when monocular views are discrepant may have better sensitivity to PC-biased stimuli but not to those biased for MC-processing. However, our present design does not provide the means to fully differentiate between anatomical or neural mechanisms for the differences. We certainly agree with Porac and Coren [47] in that ocular dominance may sometimes simply be the result of “physiological or refractive superiority.” A more modern investigation of visual evoked potential (VEP) and refractive error in 1771 anisometropic myopes revealed less ocular dominance in more myopic eyes but no higher order (i.e., VEP) differences [48]. However, neither our inclusion criteria for visual acuity nor methods were able to detect threshold level interocular differences in spatial vision. We have suggested more sensitive follow-up investigations that do so. We also make no claim that the inferred superior function for PC-biased stimuli drives preference when only a single eye can be used (as in the “hole in the card” method of sighting dominance). Rather, we suggest that improved discernibility of PC-biased stimuli may simply be the result of consistent preference for the dominant eye and suppression of the non-dominant eye. In a sense, individuals with strong dominance—as in this study—develop a subclinical amblyopia in the non-dominant eye. At least two prior investigations support this suggestion and further implicate a dominance plasticity like that of refractive amblyopia [49,50]. As we have suggested, subsequent studies should further isolate the PC and MC pathways to confirm the dominance plasticity of the separate parallel pathways.
